# A Framework for an Institutional High Level Security Policy for the Processing of Medical Data and their Transmission through the Internet

**DOI:** 10.2196/jmir.3.2.e14

**Published:** 2001-04-06

**Authors:** Christos Ilioudis, George Pangalos

**Affiliations:** ^1^Aristotle University of ThessalonikiThessalonikiGreece

**Keywords:** High Level Security Policies, Internet Security, Security of Health Care Information

## Abstract

**Background:**

The Internet provides many advantages when used for interaction and data sharing among health care providers, patients, and researchers. However, the advantages provided by the Internet come with a significantly greater element of risk to the confidentiality, integrity, and availability of information. It is therefore essential that Health Care Establishments processing and exchanging medical data use an appropriate security policy.

**Objective:**

To develop a High Level Security Policy for the processing of medical data and their transmission through the Internet, which is a set of high-level statements intended to guide Health Care Establishment personnel who process and manage sensitive health care information.

**Methods:**

We developed the policy based on a detailed study of the existing framework in the EU countries, USA, and Canada, and on consultations with users in the context of the Intranet Health Clinic project. More specifically, this paper has taken into account the major directives, technical reports, law, and recommendations that are related to the protection of individuals with regard to the processing of personal data, and the protection of privacy and medical data on the Internet.

**Results:**

We present a High Level Security Policy for Health Care Establishments, which includes a set of 7 principles and 45 guidelines detailed in this paper. The proposed principles and guidelines have been made as generic and open to specific implementations as possible, to provide for maximum flexibility and adaptability to local environments. The High Level Security Policy establishes the basic security requirements that must be addressed to use the Internet to safely transmit patient and other sensitive health care information.

**Conclusions:**

The High Level Security Policy is primarily intended for large Health Care Establishments in Europe, USA, and Canada. It is clear however that the general framework presented here can only serve as reference material for developing an appropriate High Level Security Policy in a specific implementation environment. When implemented in specific environments, these principles and guidelines must also be complemented by measures, which are more specific. Even when a High Level Security Policy already exists in an institution, it is advisable that the management of the Health Care Establishment periodically revisits it to see whether it should be modified or augmented.

## Introduction

A High Level Security Policy (HLSP) is a set of high-level statements intended to guide Health Care Establishment (HCE) personnel who are involved in the processing and management of sensitive health care information. It provides a set of mandatory regulations to ensure adequate security of personal health information processed by health information systems. "High level" in this context means that the HLSP states *what* should be done to implement security efficiently; however, it does not provide technical details on *how* to do this.

We have previously reported in detail the set of acceptable technical measures that are needed to implement an Internet security policy and we have classified them into categories, such as: encryption approaches, Web server usage, mail usage, and protection from virus and interactive software [1]. This paper defines a suitable HLSP for Health Care Establishments and establishes the basic security requirements that must be addressed in order to use the Internet to safely transmit patient and other sensitive health care information.

The Internet provides unprecedented opportunities for interaction and data sharing among health care providers, patients, and researchers. However, the advantages provided by the Internet come with a significantly greater element of risk to the confidentiality and integrity of information [2]. It is therefore essential that the Health Care Establishments develop and implement an appropriate HLSP for processing medical data and transmitting this data through the Internet.

The HLSP should be used as a reference for a wide variety of information security and privacy activities, including establishing user access privileges, and investigating security and privacy threats. The HLSP refers primarily to the people involved (including patients, doctors, administrators, and health care authorities) and the data objects that should be protected (including medical records and communication data). The HLSP should be fully adopted to be effective; in addition, conformance to its regulations should be made mandatory for all members of staff.

This work has taken place in the context of the Intranet Health Clinic (IHC) project, which is an international project involving EU (European Union) member countries and Canada. The IHC concerns a deployment of a secure Internet-based application for patient care using Internet-based advanced multimedia techniques. The aim is to offer users of health services high-quality care over inexpensive communication pathways, using Internet-based, interactive communication tools. IHC addresses patients' needs in three key health domains (oncology, respiratory diseases, and obstetrics/gynecology), along with Canadian rheumatic-disease patients, as they seek health services in a complex regional environment of large tertiary-level hospitals, secondary-level hospitals, remote primary-level health care centers, and homes. The IHC is intended to help patients discharged from a tertiary-level health care organization (eg a highly specialized hospital) who must be effectively followed-up by the primary-level physician in a geographically remote area like the many small isolated islands of Greece.

The IHC services involve image and audio transmission, Web-based education, and Intranet-multimedia patient records. The users of the application are patients and their family members, and health professionals at all levels of health care delivery (primary, secondary, and tertiary care).

## Methods

Security in health care automated information systems can be conceptually viewed at four distinct levels of abstraction: *generic principles*(that are society-dependent and culture- dependent); *principles*(that are administration-dependent); *guidelines*(that are technology-dependent) and *measures*(that are installation-dependent). The HLSP addresses the two middle levels of abstraction: *principles* and *guidelines*. Thus, an HLSP depends on generic principles and must be complemented by measures [3].

The HLSP in this document provides a set of mandatory regulations to ensure adequate security of personal information processed by the health care information systems. We developed the proposed HLSP by a top-down approach. More specifically, *principles* were first derived as a result of: considering what the functional model of a secure Health Care Establishment should be; analyzing and adapting relevant similar efforts of international bodies from the EU, USA, and Canada; and consulting with Health Care Establishment users in the context of the IHC project mentioned above [2]. Then, *guidelines* were developed, by detailing principles.

In addition to our own work and experience [2,3,4,5], the proposed HLSP has also been based on a detailed study of the related recommendations from various significant security and standard groups, mainly from the EU countries, USA, and Canada. More specifically, this paper has also taken into account major directives, technical reports, recommendations, and specific descriptions that are related to the protection of individuals with regard to the processing of personal data, the protection of medical data, and the protection of privacy and medical data on the Internet [6,7,8,9,10,11,12,13,14,15,16,17,18,19].

The proposed principles and guidelines have been made as generic and open to specific implementations as possible, to provide for maximum flexibility and adaptability to local environments. It is clear however that these principles and guidelines can only serve as reference material for developing an appropriate HLSP in a specific implementation environment. When implemented in specific environments, these principles and guidelines must also be complemented, as seen earlier, by the appropriate measures, which are installation dependent.

## Results

The result is the proposal of a suitable HLSP for Health Care Establishments. The proposed HLSP includes a set of 7 principles and 45 guidelines, which are presented below.

### 1. Limited Data Circulation Principle

**P1. All personal health data are considered sensitive and should be protected with care. Circulation of personal health data should be according to the regulations set out in the Health Care Establishment.**

*Related Security Guidelines*

G1.1. Purpose

The circulation of personal health data should take place only for Health Care Establishment purposes.

G1.2. Informed consent

The explicit and informed consent, written or recorded, of the data subject is mandatory for the disclosure of named data about this patient.

G1.3. Data release for research purposes

The release of health data for research purposes should be non-identifiable with a patient.

G1.3. Data confidentiality

All Health Care Establishment users should ensure that, in any dealings with the media, the patient's right to data confidentiality is fully safeguarded and that the patient's free and informed consent is always obtained prior to any release of the data to the media.

G1.4. Personal health data transmission

Personal health data transmission should be provided only when necessary and only for purposes of the Health Care Establishment.

G1.5. Health data storage limitation

Personal health data should be kept in a form that permits identification of the patient concerned for no longer than is necessary for the purpose for which the data are stored; when the purpose no longer exists, the data should be erased.

G1.6. Data release for educational purposes

The release of health data for educational purposes should be non-identifiable with a patient.

### 2. Security Regulations Principle

**P2. Appropriate measures should be taken for the security of health data and for the protection of the privacy of the patients, aiming at preventing:**

- denial of the services of the system,
- accidental or deliberate destruction of data,
- unauthorized access to, or disclosure of, data,
- accidental or deliberate alteration of data,
- unauthorized creation of data.

These measures comprise technical, organizational, personnel management (procedural), and physical security measures.

*Related Security Guidelines*

G2.1. Data categorization

Personal health data should be characterized within general categories, according to the security requirements of the data.

G2.2. Identifiable users

Each Health Care Establishment user needs to be recognized and identified by the user's name and function so that any patient receiving hospital care and all users of the health care information system can recognize the person to whom they transmit data, or from whom they receive data, or to whom they pass control of information systems.

G2.3. Health data integrity

Technical experts should ensure the integrity of personal health data. The use of integrity mechanisms, such as checksums, can guarantee that data have not been altered or destroyed in an unauthorized manner.

G2.4. Organizational issues

The regulations should include articles applicable to the organization and staff, such as:

- the obligation for computer staff to comply with their professional code of conduct and with the sanctions applicable in the event of non-compliance,
- designation or appointment of one person for each Health Care Establishment, with responsibility for the application of the data security principles and guidelines,
- appointment of a person responsible for data security in operations, programming, communication, filing, and similar areas (this person is not necessarily different from the one mentioned in the previous item in this list).

G2.5. Staff reminders

The security regulations should remind staff of patient's rights regarding the circulation of personal health data.

G2.6. Separable data

The medical records must be designed to enable the separation of data according to their nature (identifiers, administrative data, medical data, and demographic data), in a logical fashion.

G2.7. Secure transmission

Methods ensuring an appropriate level of security should be chosen for the transmission of personal health data within the Health Care Establishment Intranet.

G2.8. Access-rights limitation

The basic principles governing access to personal health data are the need-to-know requirements.

G2.9. Limited access

The number of user categories, in the Health Care Establishment information system, having access to personal health data should be limited to the minimum.

G2.10. Time-limited and place-limited operations

For each access profile there should be specified the associated operations that are possible (including validation, visual display, printing, copying, and statistical processing), the location within which certain of the associated operations may be carried out, and the period within which or deadline before which certain of the associated operations may be carried out.

G2.11. Access-rights procedure

Procedures providing for restrictions on access in time and space should exist. If the means for implementing these restrictions is an access-rights table of the Health Care Establishment user categories, then this should be established according to the specialty, function, job domain, hierarchy position, and intent of each user category, in connection with the category of data that is intended to be accessed.

G2.12. Monitoring facility

Computerized health information systems should record each access to the Health Care Establishment information system and have an appropriate facility to monitor details of, for example: user, date, time and place of access, operation, and nature of information.

G2.13. Improvement of regulations

The security regulations should include procedures for following-up, monitoring, and improving them.

G2.14. Encouraging security improvement

Trials of technology, software, and applications for protecting security and privacy should be supported.

G2.15 Documented security measures

A detailed description covering all the technical aspects of security of the Health Care Establishment information systems, both from a physical and a logical point of view, and all existing security procedures in force, should be documented in detail and made available to the Health Care Establishment sites.

G2.16. Security policy

A health-data technical security policy should be adopted by each Health Care Establishment site. The policy should be concerned with confidentiality, integrity, and availability of the data, as well as with accuracy, reliability, performance, and functional correctness of the information system.

G2.17. Definition of the ultimate purpose

The regulations should include the ultimate purpose of any information system that functions within the Health Care Establishment, and the type of data that it contains.

G2.18. Database security

For storing personal health data in database environments, a database-specific security policy should be established. This policy should state which kind of communication channels between users can be established, requirements for the availability of certain facilities of these channels, and requirements for the separation and non-interference of these channels.

G2.19. Teleconference Service Security

This policy should state which kinds of data are permitted to travel through teleconference services. In addition, the requirements of confidentiality and of user identification must be satisfied.

### 3. Patient's Rights Principle

**P3. Information systems in the health care field exist and operate to serve patients according to human rights and freedoms and according to constitutional provisions pertaining to civil rights. These rights are consistent with national law, but may be additional to rights embodied in it.**

*Related Security Guidelines*

G3.1. Purpose

All regulations, policies, and measures about the preservation of security of personal health data should respect human rights and freedoms, and the pertinent constitutional provisions. In no case may these rights be neglected while enforcing any security-related function.

G3.2. Knowledge of stored health data

The patient has the right to obtain, at reasonable intervals and without excessive delay or expense, confirmation of whether that patient's personal health data are stored in a file. The patient has the right to be given such data in a form that is intelligible to the patient.

G3.3. Knowledge of a processing operation

The patient has the right to know of the existence of a processing operation, its purposes, the categories of data concerned, and any third parties or categories of third parties to whom the data are to be disclosed.

G3.4. Processing of health data

The processing of personal health data should be, in principle, viewed and treated as an exceptional means to obtain information. Whoever asks for such data should be obliged to explain the need for the data: why and to what extent particular purposes cannot be fulfilled by using other information.

### 4. Health Care Service Providers' Obligations Principle

**P4. Service providers in Health Care Establishments exist, operate, and have responsibilities according to the law and according to the regulatory security framework.**

*Related Security Guidelines*

G4.1. Proper use of data

Health Care Establishment providers are responsible for proper use of data. They should declare: the kind of data they collect, process, and store; and the way of and purpose for collecting, processing, and storing the data. In addition, the introductory page of the data must have a clear statement about privacy policy.

G4.2. Technical and organizational measures

Health Care Establishment providers must take the appropriate technical and organizational measures to protect personal data against accidental or illegal destruction, accidental loss, and any form of unauthorized processing (including access, alteration, and communication).

Such measures shall ensure an appropriate level of security taking account, on the one hand, of the technical state of the art and, on the other hand, of the sensitive nature of medical data and the evaluation of potential risks.

These measures shall be reviewed periodically.

G4.3. Data separation

In order to develop effective security policy, the information produced or processed by an Health Care Establishment must be separated into: identifiers and data relating to the identity of individuals, administrative data, medical data, and demographic data.

### 5. Quality of Health Data Principle

**P5. Personal health data should be processed in a way that ensures a high quality of integrity and accuracy.**

*Related Security Guidelines*

G5.1. Accuracy

Personal health data should be accurate and, where necessary, kept up to date; every step must be taken to ensure that data that are inaccurate or incomplete, for the purposes for which they were collected, are erased or corrected.

G5.2. Protection responsibility

The Health Care Establishment is responsible for maintaining the integrity and correctness of personal health data so that it is free from both accidental and malicious errors.

G5.3. Quality evaluation

Measures should be specified to ensure the regular evaluation by Health Care Establishment staff of the quality of the software used.

### 6. Medical and Epidemiological Research Principle

**P6. Requests for health data identifiable with a person - and for a purpose previously unspecified - can be addressed, if the informed and freely-given consent of the person concerned has been obtained and if the person has been informed of rights of refusal, access, and correction.**

*Related Security Guidelines*

G6.1. Purpose

Medical and epidemiological research promotes human knowledge, thereby improving the quality of health care; therefore, epidemiological research should be encouraged, stimulated, and promoted as strongly as possible. However, preservation of confidentiality and respect for patient's rights should take precedence over any scientific purpose. Thus, release or disclosure of personal health data should be made only when specific predetermined regulations are observed.

G6.2. Erasure of research data

The patient has the right to obtain correction of inaccurate or incomplete personal health data, or the erasure or blocking of such data.

G6.3. Anonymity

Personal health data to be used for research purposes should be anonymous.

G6.4. Communication of research data

Personal health data processed for a medical or an epidemiological research project should neither be used nor disclosed for another research project or for other purposes.

### 7. Transmission of Sensitive Health Care Data over Internet Principle

**P7. Sensitive Health Care Establishment information sent through the Internet must be accessed only by authorized people. The Internet can be used for the transmission of sensitive health care data, provided that: a suitable Internet Security Policy is in place, an acceptable method of encryption is utilized to provide for confidentiality and integrity of this data, and suitable authentication or identification procedures are employed to assure that both the sender and recipient of the data are known to each other and are authorized to receive and decrypt such information.**

*Related Security Guidelines*

G7.1. Acceptable technologies

To make the Internet adequately safe for Health Care Establishments (that is, to ensure that data travel safely through the Internet, are only disclosed to authorized parties, and are not inappropriately disclosed or modified) technologies must be used that allow users to prove they are who they say they are (identification and authentication) and allow the organized scrambling of data (encryption).

G7.2. Encryption

To make the Internet adequately safe for Health Care Establishments, a complete Internet communications implementation must include adequate encryption. Encryption must be at a sufficient level of security to protect against the cipher being readily broken and the data compromised. The length of the key (a secret value used to encrypt and decrypt messages) and the quality of the encryption framework and algorithm must be increased over time, as new weaknesses are discovered and as processing power increases.

G7.3. Authentication and Identification

To make the Internet adequately safe for Health Care Establishments, a complete Internet communications implementation must employ authentication or identification of communications partners. Public key certificates can facilitate authentication and identification services through the Internet.

G7.4. Integrity

To make the Internet adequately safe for Health Care Establishments, they should be required to be able to provide corroboration that data have not been altered or destroyed during transmission through the Internet.

G7.5. Availability

To make the Internet adequately safe for Health Care Establishments, a complete Internet communications implementation must include adequate security measures to improve availability of Internet services. Information should be available when needed at appropriate places and Health Care Establishment information systems have to be protected from denial-of-service attacks.

G7.6. Non repudiation

To make the Internet adequately safe for Health Care Establishments, a complete Internet communications implementation must include adequate security measures to improve non-repudiation, so that responsibility for actions cannot be denied. These measures support the provision of evidence that will prevent a participant in an action from convincingly denying responsibility for the action.

## Discussion

This paper defines a suitable High Level Security Policy (HLSP) for Health Care Establishments and proposes the basic security requirements that must be addressed to use the Internet to safely transmit patient and other sensitive health care information. It has been based on a detailed study of the related recommendations from the more-significant security and standard groups, mainly from the EU countries, USA, and Canada. These recommendations are related to: the protection of individuals with regard to the processing of personal data, the protection of medical data, and the protection of privacy and medical data on the Internet. Therefore, the proposed HLSP satisfies the security requirements that originate from European Law and from other international recommendations. During the development of the proposed HLSP, we considered draft laws and prestandards, to achieve a state-of-the-art security policy.

There are two different security frameworks from the EU and Canada. Since these two regions have different legal frameworks, technological developments, and levels of users' concern about the security of medical data transmitted through the Internet, the proposed HLSP has an advantage. Works corresponding to the proposed HLSP include ISHTAR and Health Level Seven (HL7) security policy.

The HLSP is primarily intended for large Health Care Establishments in Europe, USA, and Canada. It should be fully adopted to be effective and conformance to its principles and guidelines should be made mandatory for all members of staff. Even when an HLSP already exists, it is advisable that the management of the Health Care Establishment periodically revisits the HLSP to see whether it should be modified or augmented.

Currently, there is no specific national law on the protection of privacy and medical data on the Internet. We expect that in the future there will be important laws and recommendations that will affect the protection of medical data transmitted through the Internet.

## Abbreviations

EU: European Union
HCE: Health Care Establishment. An establishment where medical services are provided or health education, research, medical-training or prevention activities are conducted.
HLSP: High Level Security Policy. A set of high-level statements intended to guide those members of the Health Care Establishment personnel who are involved in the processing and management of sensitive health care information.
IHC: Intranet Health Clinic project. A deployment of a secure Internet-based application for patient care, using Internet-based advanced multimedia techniques.
